# Black Truffle Aroma Evaluation: SPME-GC-MS vs. Sensory Experts

**DOI:** 10.3390/foods13060837

**Published:** 2024-03-09

**Authors:** Eva Tejedor-Calvo, Sergi García-Barreda, Sergio Sánchez, María Ángeles Sanz, Pedro Marco

**Affiliations:** 1 Department of Plant Science, Agrifood Research and Technology Centre of Aragon (CITA), Av. Montañana, 930, 50059 Zaragoza, Spain; sgarciaba@cita-aragon.es (S.G.-B.); pmarcomo@cita-aragon.es (P.M.); 2 Agrifood Institute of Aragón—IA2, University of Zaragoza—CITA, 50018 Zaragoza, Spain; 3 Laboratory for Aroma Analysis and Enology, Aragón Institute of Engineering Research (I3A), Department of Analytical Chemistry, Faculty of Sciences, University of Zaragoza, 50009 Zaragoza, Spain; 4 Laboratories and Technological Assistance, Agrifood Research and Technology Centre of Aragon (CITA), Avda. Montañana, 50059 Zaragoza, Spain

**Keywords:** *Tuber melanosporum*, black truffle, trained panel, volatile organic compounds, sensory analysis

## Abstract

Nowadays, the truffle aroma attribute is not included as a quality parameter in the current recommendation that explains the truffle quality (UNECE standard 53 FFV3) and establishes the truffle commercial categories. However, the aroma is the main reason why truffles are worldwide appreciated. Indeed, more than 30 aromatic molecules compose it, and this is the reason why the human evaluation and identification of these odorants, without previous training, is quite subjective. Analytical techniques such as gas chromatography techniques, however, can establish an aromatic profile and detect potential aromatic markers. In this study, 16 tasting experts were trained to make more objective the truffle aroma evaluation and odorants identification. For this, a comparison between solid-phase microextraction gas chromatography coupled with mass spectrometry (SPME-GC-MS) and sensory expert evaluation was carried out in six sessions during different harvesting times in the black truffle season (December, January, and February). Both techniques were able to separate truffles depending on the harvesting time. Also, a list of volatile organic compounds related to the aromatic attributes was reported. This information will help to provide a more objective *T. melanosporum* truffle sensory evaluation.

## 1. Introduction

The black truffle (*Tuber melanosporum* Vittad.) is recognized internationally thanks to its organoleptic properties, especially the aroma. It is composed by more than 200 volatile molecules [1,2,3,4,5], which attribute a very complex and singular odor and grant truffles delicacy and gourmet properties. Some of the compounds described such as dimethyl-sulfide (DMS), dimethyl disulphide (DMDS), 2,3-butanodione, ethyl 2-methylbutyrate, ethyl 3-methylbutyrate, 1-octen-3-one, 2-acetyl-1-pyrroline, acetic acid, methional, (E, Z)-2,6-nonadienal, (E, Z)-2,4-nonadienal, and 3-ethylphenol have aromatic qualities [6,7]. The attributes sulfur, mushroom, earthy, butter, black olives, leather–animal, blue cheese, nuts, and alcohol have been previously described as aromatic notes to describe and evaluate the black truffle odor [8].

Aromatic properties evaluations are usually performed using analytical instruments since odor determination by humans is subjective. However, the International Organization for Standardization (ISO) standards 13301:2002 (general guidance for measuring odor, flavor, and taste detection thresholds via a three-alternative forced-choice procedure) [9] and 5496:2006 (initiation and training of assessors in the detection and recognition of odors) establish specific guidelines to train and detect food aromatic properties [10], and therefore a more objective evaluation could be carried out. These two guidelines describe the key to recognize, learn, and evaluate odor attributes in general. But, their application to black truffle aroma evaluations is necessary before considering the aroma as a quality parameter.

A variety of analytical methods are commonly used for analytical aromatic compounds determination [11]. One of the most common methods used to extract the aromas is the solid-phase microextraction (SPME) technique. The principle of this sampling technique is to reach an equilibrium state in which the concentrations of volatiles are equilibrated between truffle samples and the gaseous phase above. At this equilibrium stage, the extraction of volatiles is performed exposing an SPME fiber in the head space of the sample for a specific time (usually 10–30 min) and at a specific temperature. Different types of SPME fibers have been used for truffle aroma extraction. However, 50/30 µm divinylbenzene/carboxen/polydimethylsiloxane (DVB/Car/PDMS) is the most used [7,8,12,13,14,15]. Once the volatile compounds are attached into the fiber, its content is analyzed via gas chromatography (GC) coupled with mass spectrometry (MS) or olfactometry (GC-O) [7,8]. Both techniques are based on GC; the only difference between them is the detector used. MS has higher sensitivity, specificity, and accuracy for the analysis of VOCs compared to the flame ionization detector (FID) usually used in the GC-O technique.

Nowadays, truffle commercialized categories are regulated by the marketing and commercial quality control of truffles standard (United Nations Economic Commission for Europe (UNECE) Standard FFV-53, United Nations, 2017) [16]. However, this is just a recommendation, not a mandatory norm that explains how to classify the truffle quality. This recommendation only classifies truffles according to their weight, morphological, and physical aspects in extra, first, and second classes. It also associates the scientific name of the different truffle species with their common names and defines the quality requirements for truffles after preparation and packaging. However, the aromatic properties, the most valued attribute in truffles, are not considered in any standard or legislation. Due to the highly specialized devices and knowledge required for chromatography and olfactometry, these methodologies are not quick and cheap to implement for truffle quality inspections in local fairs, cooking competitions or protected geographical indication schemes. A trained panel with an ability to discriminate truffle aromas similar to that of analytical methods might be considered as an objective tool to evaluate truffle aroma and therefore help us consider aromatic properties a key feature in commercial truffle quality assessments. Indeed, some studies have proved a similar sensory evaluation ability between the gas chromatography method and a trained panel in coffee [17], strawberry [18], and apple juice [19], as well as in truffles [8].

The aim of this study is to compare SPME-GC-MS profiles of fresh black truffles with an evaluation made by a group of trained sensory experts. For this, fresh black truffles were simultaneously evaluated via the SPME-GC-MS technique and by the trained panel in six different sessions throughout the harvesting truffle season in Spain (season 2022–2023). It is expected that the results will provide what attributes have to be evaluated in black truffle sensory training as well as the evidence that a trained panel can discriminate black truffle samples like the gas chromatography method.

## 2. Materials and Methods

### 2.1. Sampling Material

*Tuber melanosporum* ascocarps were harvested in various truffle orchards of Teruel, Zaragoza, and Castellón provinces (eastern Spain) throughout the harvesting season (December to February). The climate is continental Mediterranean, with a mean annual rainfall of 519 mm and a mean annual temperature of 11.1 °C, typical of Spanish truffle-producing regions [20]. This is important because, in early seasons, it is much more common to find commercial truffles with a lower maturity (i.e., lower spore maturity and pigmentation, which is linked to lighter gleba colors) and ripeness (i.e., an aroma that has not achieved the typical complexity and intensity of fully ripe truffles).

All truffles selected were characterized by the typical black truffle aroma, and the maturity stage of the truffles was assessed with a gleba sample reaching 5–10 mm under the peridium, taken using a scalpel. With this sample, a spore maturity index was calculated as the percentage of asci containing mature (i.e., dark brown and spiny) spores [21]. Fresh truffles (between 50 and 80 g each) were identified, selected, and processed [1]. All the following analysis were carried out the same day or the day after harvesting the ascocarps. The experiment was conducted during the truffle season (two sessions in December 2022, January 2023, and February 2023).

### 2.2. Volatile Organic Compounds Analysis by SPME-GC-MS

The methodological approach was carried out according to Tejedor-Calvo et al. (2023) [22]. Briefly, a solid-phase microextraction (SPME) was used to extract the aromatic compounds. For this, a fused silica fiber coated with a 50/30 µm layer of DVB/Car/PDMS from Supelco (Barcelona, Spain) was chosen. The samples (2 g of truffle) were placed in a 20 mL glass vial closed with a cap with septum PTFE/sil. After this, the vial was conditioned at 50 °C for 10 min. The fiber was then exposed to the headspace of the vial for 20 min. Analyses were carried out by duplicate.

The volatile organic compounds (VOCs) profile of the different samples was analyzed via GC–MS using a gas chromatograph Agilent 6890 series coupled with a mass selective spectrometer detector 5973N series (Agilent Technologies, Santa Clara, CA, USA). This instrument was equipped with a capillary column HP-5MS (Agilent Technologies, Santa Clara, CA, USA) of 30 m, 0.25 mm i.d., 0.25 μm film thickness and a flow of 1 mL/min with helium as the carrier gas. The injector temperature was 250 °C. The SPME fiber was injected and desorbed during the first 5 min of the running. The oven temperature was 45 °C, which was held for 2 min, 45–246 °C at a rate of 5 °C/min, and finally to 250 °C at 10 °C/min and held for 4 min. The MS used the electron impact mode with an ionization potential of 70 eV and an ion source temperature of 230 °C. The interface temperature was 250 °C. The MS scanning was recorded in full-scan mode (35–350 *m*/*z*). MSD ChemStation (Agilent G1701DA) software was used for controlling the GC–MS system.

Peak identification of the VOCs was achieved via a comparison of the mass spectra with mass spectral data from the Wiley275 and NIST MS Search Program 2.0 libraries and via a comparison of previously reported retention indexes (RIs) with those calculated using an n-alkane series (C6–C20) (purity 99%) (C6, C7 Merck, C8–C20 Supelco, Merck KGaA, Darmstadt, Germany) under the same analysis conditions. Truffle composition in percentage was calculated according to the area of the peaks in total ion chromatogram (TIC) mode.

### 2.3. Sensory Analysis by Trained Panel

#### 2.3.1. Experts Selection and Training

A panel of sixteen trained tasters (23–55 years old; seven males and nine females) who had previously participated in truffle evaluations [8] were selected. Tasters were specifically trained about truffle aroma compounds, ISO 13301:2022 (sensory analysis: general guidance for measuring odor, flavor, and taste detection thresholds by a three-alternative forced-choice procedure) [23] and 5495:2006 (sensory analysis: initiation and training of assessors in the detection and recognition of odors) [24] in four sessions of one hour. The panelists gave their consent to take part and for us to use their information, and the appropriate protocols for protecting the rights and privacy of all participants were used. Also, the Agrifood and Technological Research Centre gave us permission to conduct sensory panel research.

After the formation, an evaluation of six truffles was carried out by triplicate in three different sessions (two truffles per session). This experiment was carried out in order to check the agreement among assessors (agreement ability of different assessors to exhibit the same product differences when assigning scores on a given attribute to the same set of products) [21], evaluate possible attribute or assessor effects, and evaluate possible correlations among aromatic attributes (e.g., if a truffle smells like mushroom, this shows a lower sulfur aroma).

The trained panel analyses were conduct according to the ISO 11035:1994 (Identification and selection of descriptors for establishing a sensory profile by a multidimensional approach) [25], and the following aromatic parameters were selected according to previous studies [6,8]: sulfur, black olives, mushroom, leather, fermentation, nuts, straw, equilibrium, intensity and complexity. Each parameter was assessed with a 9-point rating scale (1 minimum and 9 maximum).

#### 2.3.2. Expert Evaluation

Once the trained panel was calibrated, a total of 14 samples were evaluated in six sessions (2–4 truffles per session to avoid oversaturation; by 16 truffle experts). The evaluation consisted in two phases: one for visual attributes and one for olfactive attributes. The following attributes were evaluated in the visual phase: firmness, shape (roundness), uniformity (lobularity), peridium color, peridium shape, gleba color, and sharpness of the marbled pattern of the gleba (Table S1). Each parameter was assessed with a 9-point rating scale (the scale was established with the experts for each attribute) [8]. The attributes for the olfactive phase were the same used during the panel training. The sensory analysis was carried following ISO Norm 8589:2007 (General guidance for the design of test rooms) [26]. The temperature of the room was about 21–22 °C.

Firstly, the visual phase was evaluated using a whole ascocarp. Then, a slice was cut for the aromatic phase (2 mm thickness with a steel laminator). To avoid oxidation, the olfactive phase was carried out in two minutes. In case the aroma changed (more than two minutes of exposition) during the tasting, another slice was cut. Since fresh truffle has a very short shelf life (approx. 14 days) and the tasting is destructive (one slice is necessary to detect the aromas), the repetitions were carried out the same day instead of on different days.

### 2.4. Statistical Analysis

The VOCs were analyzed via principal component analysis (PCA), performed and visualized in RStudio 1 February, 1335 (RStudio Team, 2019) using R version 3.6.1 and the factoextra package (Kassambara and Mundt, 2017). Training and evaluation sensory data were analyzed via the PanelCheck program V1.4.2. The relation between the odor detected by the trained panel and aromatic VOCs was analyzed via the SankeyMATIC program.

## 3. Results and Discussion

### 3.1. Volatile Organic Compounds Analysis via SPME-GC-MS

A total of sixty-nine VOCs were detected using the SPME-GC-MS technique (Table 1). These molecules were previously found in *T. melanosporum* truffles [1,7]. Truffle maturity changes can be observed in the spore maturity [27], chemical composition [28], and bacterial community profile [29,30] throughout the season. As indicated by Caboni et al. (2020) [31], metabolite levels in truffles strongly depend on the month of harvesting which is directly related to the maturity stage. Therefore, some changes are expected in the aromatic compounds as well.

Different VOCs profiles were observed in truffle samples from different harvest times (Table 1). Truffles from December (time 1) showed a high content of butanal-3-methyl (17.7%), butanal-2-methyl (13.6%), hexanal (12.2%), DMS (9.4%), 1-octen-3-ol (6.7%), and 3-methylanisol (5.1%). In January (time 2), the dominant VOCs were very similar but with different percentages: DMS (21.8%), 2-methyl-1-butanol (17.7%), butanal-2-methyl (13.2%), metylpropylformate (10.3%), and 2-methyl-1-propanol (5.8%). At the end of the season, in February (time 3), the most abundant molecules were 2-methyl-1-butanol (29.25%), DMS (11.1%), butanal-2-methyl (8.8%), DMDS (6.3%), and 2-methyl-1-propanol (5.6%). Apart of them, some of the key truffle VOCs described for black truffle such as 3-methyl-1-butanol, 2-methyl-1-butanol, 2-butanone, and anisole [1,2,3] were also found in our samples. Some key compound contents changed their relative abundance throughout the season, i.e., butanal-3-methyl decreased from 17.7% to 4.5%, whereas 2-methyl-1-butanol increased from 3.1% to 21.3%. However, 2-propanone, butanal-2-methyl, and anisole maintained constant percentages in the aromatic profile. According to Caboni et al. (2020) [31], aromatic compounds can be used as ripening markers, and those previously described could be potential markers to establish VOCs profile for different truffle maturity stages.

**Figure 1:**
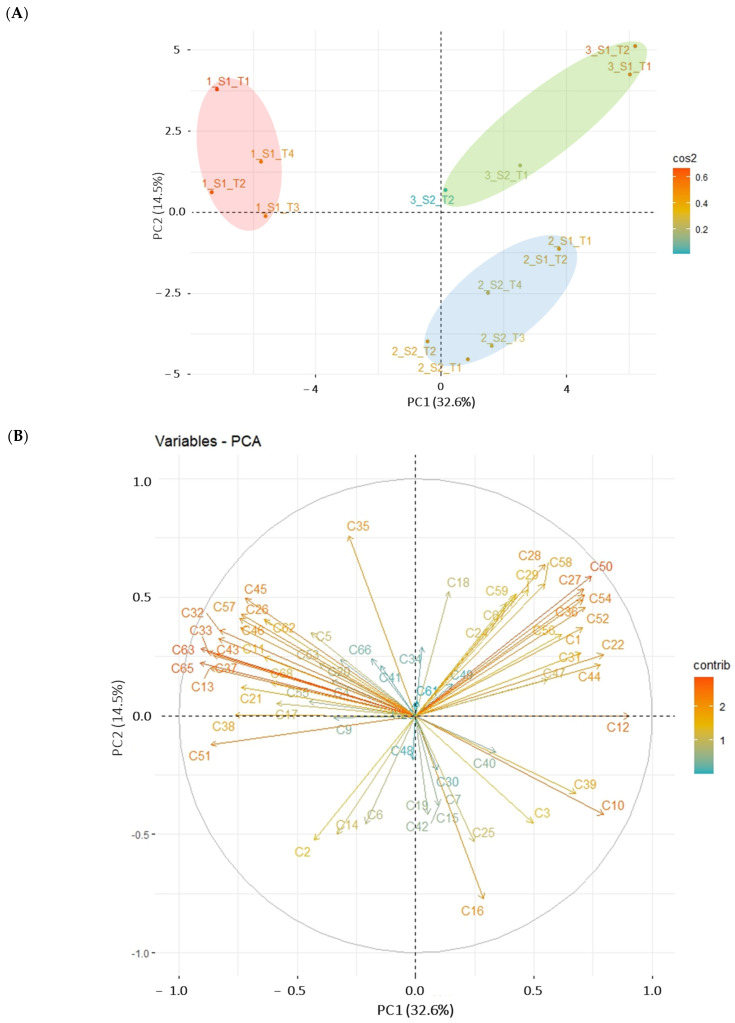
PCA results for the VOCs profile of fresh black truffles (n = 14) sampled at three different harvesting times: (A) score plot of the analyzed ascocarp samples and (B) loading plot for the VOCs detected via SPME-GC-MS. In (A), sample color indicates the quality of representation for the sample (cos2). In the sample names, the first number (1, 2, 3) refers to the month harvested: 1—December, 2—January, 3—February; S1 or S2 refer to the session number (two per month), T1–T4 correspond with the number of truffles in the session. In (B), compounds are identified with numbers corresponding to those in Table S1, and arrow color indicates the contribution of a compound to the PCA components (contrib). The 95% confidence ellipses colors correspond to the harvesting time: December (red), January (blue), February (green).

A PCA was used to explore the possible linkages between harvesting time and VOCs content detected via SPME-GC-MS (Figure 1).

The PCA explained 47.1% of the data variability with the two first PCA components. The first component allowed us to clearly separate the first harvesting time from the others, whereas the second did not explain the maturation degree which might be due to other factors. An early harvesting time was positively associated with 2-butanone (C10), 2-methyl-1-propanol (C12), 2-methyl-1-butanol (C22), 3-octanol (C39), 2-ethyl-1-hexanol (C44), isoamyl-2methylbutyrate (C50), 2-methyl-butanoic acid (C52), and benzeneethanol (C54). Some of these molecules have wine and onion aromas (2-methyl-1-butanol), a mushroom-like aroma (3-octanol), rose and green odors (2-ethyl-1-hexanol), and honey and rose odors (benzeneethanol) [32]. Other compounds such as butanal-3-methyl (C13), methional (C33), nonanal (C51), 2,5-dimethoxytoluene (C63), or 2-undecanone (C65) were positively associated with truffles harvested in January and February. Some of them show malt (butanal-3-methyl), cooked potato (methional), fat and citrus (nonanal), and orange odors (2-undecanone) [32]. This difference might be due to some degree of aromatic immaturity in December truffles with respect to those harvested in January and February. Moreover, truffle aromas can vary depending on many factors, such as bacteria community, host tree, and geographical origin, among others [33,34,35]. For example, Culleré et al. (2016) identified some markers linked to the host tree: DMS was higher in hazel and holm oak truffles, whereas isoamyl alcohol was higher in Portuguese and kermes oak [35]. Strojnik et al. (2020) reported a wide volatilome study including more than 450 truffle samples from 11 different countries. The study revealed an aromatic model able to differentiate between species with an overall classification rate of 97%. The article indicated that many factors, including genotypic variability, maturation, microbial community as well as geographical origin need to be considered for the understanding of the aromas [34]. Our results showed that SPME-GC-MS technique was able to differentiate fresh truffles depending on the harvest time during the season, even though they all showed optimal maturity according to spores and gleba color [25,36].

### 3.2. Truffle sensory Evaluation by the Trained Panel

#### 3.2.1. Training Truffle Aroma Analysis

Since aroma is one of the most difficult attributes to evaluate due to subjectivity issues, some training was necessary before starting the experiment. Each expert tested, three times, each truffle (and two truffles per session) to check the agreement among experts in the scores of the attributes.

The Tucker-1 plots (Figure S1) display how the experts were related to each other in the attributes evaluation. In these plots, the more structured information an attribute contains, the closer it appears to the outer circle (100% explained variance for that attribute). The attributes sulfur, black olives, mushroom, and leather plots showed all the expert variance in the circles except for expert 9 and 13 in mushroom and black olives, respectively. In general, these results indicate a good evaluation of these attributes. However, in those in which some experts were outside the circle, more focused training with those experts was carried out to ensure a more objective evaluation.

**Figure 2:**
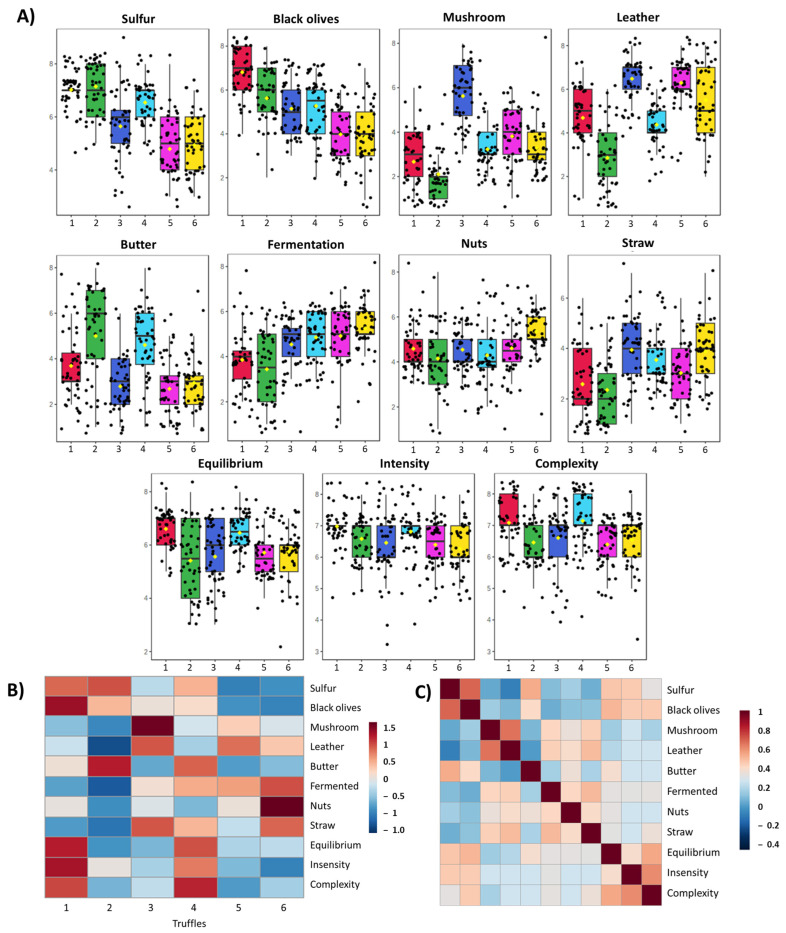
Sensory panel training data: (**A**) sensory attribute scores of six truffles (named from 1 to 6 and shown in different colors); (**B**) heatmap of sensory profile of each truffle sample characterized by the trained panel; (**C**) correlation among sensory attributes evaluated by the trained panel during the training. Data from correlation and *p*-value are shown in Table S2. Correlation values higher than 0.116 in absolute value are significant at alpha = 0.05 level (n = 288).

In order to assess the relevance of the odor descriptors and their relationships, an in-depth analysis of the aromatic attributes was conducted (Figure 2). Despite the between-truffles variability in the scores of the olfactive attributes, in general, sulfur, black olives, leather, and butter showed the highest scores. Fermentation, nuts, and straw attributes showed a more constant and lower score evaluation throughout samples. Also, equilibrium, intensity, and complexity score were high in all the samples, indicating that fresh truffles usually have a strong and complex aroma (Figure 2A).

A heatmap was used to visualize the aromatic profile of each truffle (Figure 2B). Truffles 1, 2, and 4 showed a similar profile with high intensities in sulfur, black olives, and butter attributes, whereas truffles 3, 5, and 6 reported higher intensities in mushroom and leather attributes. Truffles 1 and 5 seemed to have more intense and complex aromas, and this could be correlated with the black olives aroma content or with the combination of several aromatic attributes. The possible correlation among aromatic attributes was analyzed (Figure 2C; Table S2). A high correlation among sulfur and black olives attributes (r = 0.583, *p* < 0.001, n = 288) was observed, as well as among the mushroom and leather combination (r = 0.545, *p* < 0.001, n = 288). The aromatic attribute butter showed a good correlation with sulfur (r = 0.370, *p* < 0.001, n = 288) and black olives (r = 0.195, *p* < 0.001, n = 288) but negative with mushroom (r = −0.254, *p* < 0.001, n = 288) and leather (r = −0.361, *p* < 0.001, n = 288).

These results indicate that truffle aromas might have two different profiles: profile 1 with sulfur, black olives, and butter aromas and profile 2 with mushroom and leather notes. The attributes fermentation, nuts, and straw were positively correlated with the second profile and negatively with the first one. The complexity was also correlated with equilibrium (r = 0.385, *p* < 0.001, n = 288) and intensity (r = 0.472, *p* < 0.001, n = 288). Despite the fact that the correlation of these three attributes with the two profiles was not too strong, positive values were correlated with profile 1 and negative with the second. This might indicate that profile 1 is more complex and intense than profile 2. These aromatic differences might be due to several conditions such as the host tree, soil bacteria composition, or maturity. However, these truffles were harvested on the same day, with the same dog and same orchard. Therefore, the differences might be due to environmental or genetical factors [37,38].

#### 3.2.2. Fresh Truffle Evaluation

The sensory evaluation of fresh truffles was conducted by the trained panel (Table 2). The evaluation was divided into two parts: visual and olfactive phases. In the visual phase, differences among some of the samples were found for the attributes’ shape and uniformity, with sample 3_S1_T1 showing scores significantly lower than samples 1_S1_T1 and 3_S1_T2 (Table 2). These results indicate that the trained panel was able to discriminate truffles using these attributes with low deviation (0.7–1.3). No significant differences were found for the attribute firmness, peridium color, and sharpness of the gleba pattern, which the experts were not able to discriminate among the truffles (Table 2). In the olfactive phase, the attributes’ equilibrium, intensity and complexity showed no significant difference in the truffles studied (Table 2). Differences among some of the samples were found for the attributes sulfur aroma and mushroom aroma, whereas attributes such as leather, butter, fermentation, nuts, and straw did not show statistical differences (Table 2). In agreement with the results previously obtained with the training data, those samples showing highest scores in sulfur and black olives attributes reported the lowest mushroom and leather values, i.e., samples 1_S1_T3, 2_S2_T3, 3_S2_T1. Only 2_S2_T1 showed mushroom values (5.9) higher than sulfur (3.1) and black olives (3.9).

**Figure 3:**
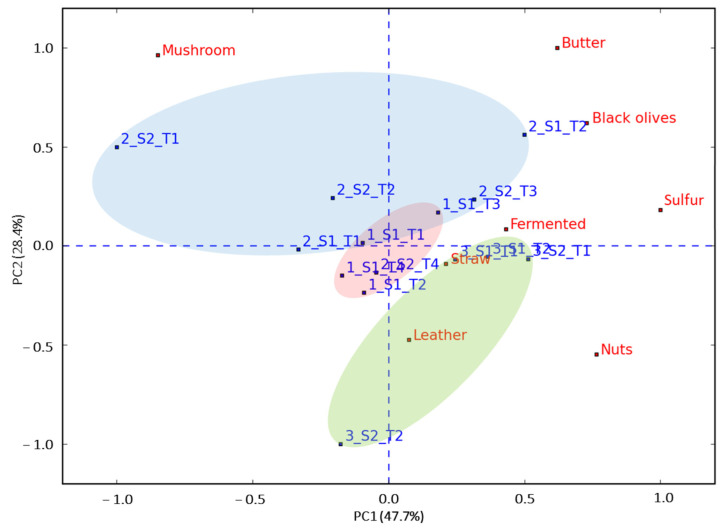
Bi-plot from correspondence analysis of evaluated aromatic attributes (colored in red) for 14 truffles samples (colored in blue) evaluated by the trained panel. Samples are labeled with the same identificatory factors as in Table 2. The 95% confidence ellipses colors correspond to the harvesting time: December (red), January (blue), February (green).

A correspondence analysis was used to explore the correlation between truffle maturity (during harvest time) and aromatic attributes evaluated by the trained panel (Figure 3). This analysis was carried out with only the aromatic attributes in order to detect similarities with the VOCs study. The PCA explained 76.1% of the variability with the first two components. The attribute that showed the most positive loading with the first PCA component was sulfur, whereas the mushroom attribute showed the most negative (Figure 3). The second PCA component clearly separated the truffle samples in three clusters corresponding to harvest time. The first harvest time (December) was associated with sulfur, fermentation, and straw aromas. Truffles from January were associated with mushroom, black olives, and butter aromas. This was the harvesting period with the highest variability among samples. The third harvest time (February) was associated with leather and nuts aromas. The latter aromatic attributes have been associated with truffles preserved via freeze-dried and canned treatments [6]. By contrast, black olives, butter, and sulfur attributes are considered key aromas for fresh black truffle [8].

### 3.3. Relationships between Sensory and Instrumental Aroma Measurements

**Figure 4:**
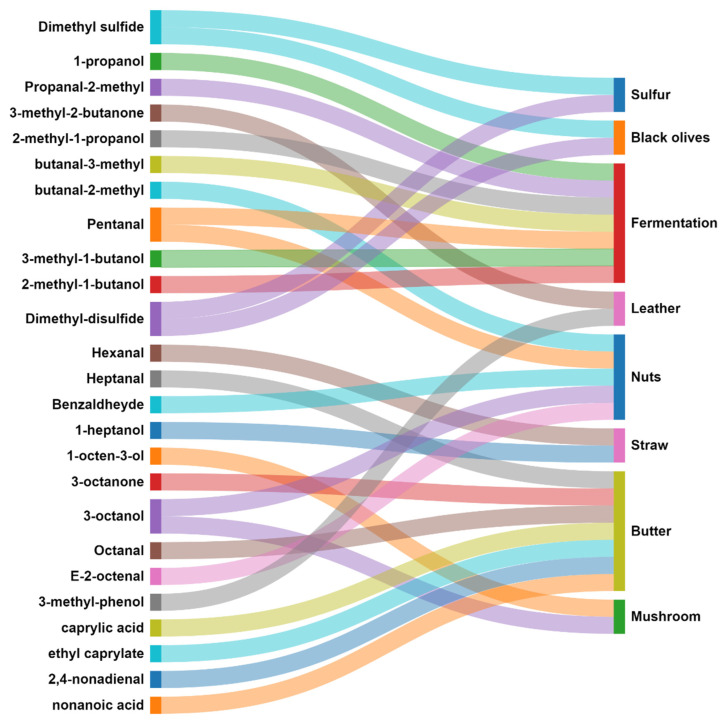
Sankey diagram of sensory attributes evaluated by the trained panel and aromatic VOCs. Data correspond to the relation established in Table S3. Each correlation was scored as 1 in the diagram.

The application of PCA analysis with VOCs and sensory data separated the samples with similar behavior, according to the harvesting time. The trained panel plot explained 76.1% of the variability with the first two components, showing a higher percentage of explained data variability compared with the VOCs PCA (47.1%). This difference can be explained by the amount of data in the VOCs PCA (69 compounds) compared to eight aromatic attributes evaluated in this PCA. A Sankey diagram was used to visualize possible relations between sensory attributes evaluated by the trained panel and aromatic VOCs (Figure 4). Only the VOCs detected by SPME-GC-MS with aromatic notes were represented in the diagram. Truffle sulfur and black olive aromas were due to DMS and DMDS compounds. Several authors agree [2,7], but more molecules have the same aromatic attribute in *T. melanosporum*. For instance, Splivallo and Ebeler (2015) [5] identified four thiopentene derivates in T. *borchii* as sulfur volatiles that contribute to human-sensed truffle aromas. Tejedor-Calvo et al. (2023) [7] reported up to three non-identified molecules with truffle odor descriptors in *T. melanosporum* via gas chromatography–olfactometry. Furthermore, Campo et al. (2017) [6] reported a non-identified molecule in black truffle with a truffle aroma using the same technology. Methanethiol has been also reported as a truffle aroma; indeed, its conversion into DMS, DMDS, dimethyl trisulphide, and H_2_S in *T. melanosporum* and T. *borchii* has been reported [4,39].

The fermentation aroma might be due to 1-propanol (alcohol, pungent), 2-methyl-propanal (pungent, malt), butanal-3-methyl (malt), pentanal (malt, pungent), 3-methyl-1-butanol (malt, whiskey), and 2-methyl-1-butanol (wine, onion). All these molecules were previously found in *T. melanosporum* [2,6,7] and are related to malt, wine, and whiskey fermentation processes. The attribute leather aroma was linked with 3-methyl-2-butanone and 3-methyl-phenol. Furthermore, 3-ethylphenol, 3-ethyl-5-methylphenol, p-cresol, and 3-propylphenol have been also reported as compounds with leather aromatic notes [2,6]. The molecules butanal-2-methyl, pentanal, benzaldehyde, 3-octanol, and E-2-octenal were related to nut odors. Also, 2-acetyl tetrahydropyridine and 2-acetyl-1-pyroline compounds were described as almond-like and toasted almond odors, respectively, in *T. melanosporum* truffles [7].

The straw aroma was linked to hexanal and 1-heptanol molecules. The first one has been also described as leafy [6]. Some identified enzymes, that play a key role in fruits, vegetables, and mushrooms were able to release some odorous compounds like green–grassy-smelling aldehydes such as hexanal [40]. This aromatic attribute might be due to a combination of other molecules since hexanal and 1-heptanol have a greener smell. Nevertheless, depending on the molecule concentration, the smell might be different [41]. The butter aroma has been related to molecules such as heptanal, 3-octanone, octanal, caprylic acid, ethyl caprylate, 2,4-nonadienal, and nonanoic acid. Also, 2,3-butadonione and 2,3-pentanodione were shown as buttery smell compounds in *T. melanosporum* and *T. aestivum* truffles [7,42]. The typical butter aroma is mainly due to lactic acid bacteria fermentation, and (E)- and (Z)-2-nonenal and (E,E)-2,4-decadienal compounds confer green and oily notes [43]. Regarding the mushroom aroma, it was associated with 1-octen-3-ol and 3-octanol among the VOCs detected. Other compounds such as 3-octanone, 1-octen-3-one, 3-octanol, Z-5-octen-1-ol, and 1-octen-3-ol were assigned as potential signaling molecules produced by truffle mycelium and fruiting bodies with a mushroom-like aroma [44]. These compounds are mainly formed by the oxidation of linoleic and linolenic acid in the presence of enzymes, such as lipoxygenase and hydroperoxidelyase [45].

The aromatic attributes proposed for the trained panel evaluation are usually a combination of some molecules, i.e., DMS and DMDS for truffle aromas. Other aromatic or non-aromatic molecules could be combined showing different aromatic signals. Furthermore, the technique used to extract the volatiles is selective in comparison with the head space technique because only some compounds were attached into the fiber. Therefore, these molecules did not attach or stick to the fiber, but with a low concentration under the analytical technique detection threshold, they were not detected and could be part of the aroma linked to the aromatic attributes selected. For this, a deeper study using different chromatographic analysis might help us understand better the truffle aroma detection by humans.

## 4. Conclusions

A black truffle (*Tuber melanosporum*) aroma evaluation was carried out using a chromatographic technique and sensory evaluation by a trained panel with similar results. Both techniques were able to separate truffles depending on the harvesting time (December, January, or February). The truffle VOCs profile showed slight differences among the seasons, reporting a higher amount of 1-octen-3-ol, hexanal, and 3-methyl-butanal at the start of the season, whereas dimethyl sulfide, 2-methyl-butanol, 2-methyl-propanol, and 2-butanone, among others, showed the highest percentages at the mid-end season. Two different truffle aromatic profiles were distinguished by the trained panel: (1) one with sulfur, black olives, and butter aromatic notes and (2) one with mushroom and leather aromas. The 69 volatile organic compounds detected were related to the following eight sensory attributes: sulfur, black olives, mushroom, leather, butter, fermentation, nuts, and straw. The use of analytical standards of the aromatic molecules related would provide help for sensory training as well as more objective truffle aroma evaluations. The results obtained in this study are the bases to develop an aromatic kit, like the existing wine or beer aromatic kits, for experts training.

## Figures and Tables

**Table 1:** List of volatile organic compounds identified via SPME-GC-MS in truffle samples and changes in their relative area (%) throughout the three harvesting times (1, 2, 3 corresponding to December 2022, January, and February 2023). Data correspond to mean ± standard deviation. Different letters within the same row (compound) denote significant differences (*p* ≤ 0.05).

						Harvest Time		
No	Name	CAS	RT	RIexp	RIlit	1	2	3
C1	ethanol	64-17-5	1.546	<600	427	0.47 ± 0.22 ^b^	1.15 ± 0.57 ^ab^	2.11 ± 1.38 ^a^
C2	2-propanone	67-64-1	1.618	<600	500	2.13 ± 0.46 ^a^	2.06 ± 0.75 ^a^	1.55 ± 0.30 ^a^
C3	dimethyl sulfide	75-18-3	1.690	<600	521	9.41 ± 4.54 ^b^	21.84 ± 3.53 ^a^	11.05 ± 6.61 ^b^
C4	methylene chloride	75-09-2	1.700	<600	531	0.31 ± 0.61 ^a^	-	0.00 ± 0.01 ^a^
C5	1-propanol	71-23-8	1.800	<600	548	-	-	-
C6	propanal-2-methyl	78-84-2	1.813	<600	560	4.19 ± 3.48 ^a^	4.97 ± 4.41 ^a^	1.77 ± 1.51 ^a^
C7	isopropyl formate	625-55-8	1.943	<600	587	-	0.32 ± 0.77	-
C8	butanal	123-72-8	1.950	<600	-	-	-	-
C9	3-methyl-2-butanone	563-80-4	1.990	<600	-	0.59 ± 1.17	-	-
C10	2-butanone	78-93-3	1.993	600	602	0.33 ± 0.65 ^b^	4.74 ± 1.36 ^a^	3.41 ± 1.05 ^a^
C11	hexane	110-54-3	1.995	600	-	0.82 ± 0.99	-	-
C12	2-methyl-1-propanol	78-83-1	2.173	620	626	0.90 ± 0.62 ^b^	5.77 ± 1.53 ^a^	5.61 ± 3.44 ^a^
C13	butanal-3-methyl	590-86-3	2.404	647	646	17.68 ± 3.17 ^a^	5.16 ± 2.80 ^b^	4.46 ± 1.17 ^b^
C14	butanal-2-methyl	96-17-3	2.512	659	653	13.63 ± 6.73 ^a^	13.28 ± 11.09 ^a^	8.83 ± 3.45 ^a^
C15	2-propanone-1-hydroxy	116-09-6	2.550	664	-	-	-	-
C16	metylpropylformate	589-40-2	2.670	678	-	-	10.34 ± 6.62 ^a^	2.87 ± 1.76 ^b^
C17	2-pentanone	107-87-9	2.728	684	687	0.46 ± 0.54 ^a^	-	0.07 ± 0.15 ^a^
C18	pentanal	110-62-3	2.730	685	704	0.10 ± 0.20 ^a^	-	0.20 ± 0.40 ^a^
C19	propylacetate	109-60-4	3.110	717	715	-	-	-
C20	3-methyl-1-butanol	123-51-3	3.485	742	737	0.46 ± 0.19 ^a^	0.21 ± 0.25 ^a^	0.46 ± 0.33 ^a^
C21	2-butanal-2-methyl	497-03-0	3.636	753	749	0.96 ± 0.65	-	-
C22	2-methyl-1-butanol	137-32-6	3.701	757	743	3.14 ± 1.59 ^b^	17.71 ± 14.4 ^ab^	29.25 ± 6.40 ^a^
C23	dimethyl-disulfide	624-92-0	3.823	765	742	0.22 ± 0.45 ^a^	0.27 ± 0.24 ^a^	6.34 ± 12.68 ^a^
C24	2-methyl-pentanal	123-15-9	3.930	772	-	-	-	0.05 ± 0.10
C25	isobutylacetate	110-19-0	4.335	800	767	-	0.13 ± 0.15 ^a^	0.03 ± 0.06 ^a^
C26	hexanal	66-25-1	5.050	817	801	12.19 ± 8.36 ^a^	1.10 ± 0.40 ^b^	1.68 ± 0.63 ^b^
C27	ethyl-2-methylbutanoate	7452-79-1	6.814	858	853	-	0.06 ± 0.09 ^a^	0.19 ± 0.16 ^a^
C28	ethyl-3-methylbutanoate	108-64-5	6.943	862	851	-	-	0.04 ± 0.05
C29	hexanol	111-27-3	7.810	882	867	0.98 ± 0.99 ^a^	1.17 ± 0.62 ^a^	1.96 ± 0.85 ^a^
C30	isoamylacetate	123-92-2	7.851	883	877	-	0.05 ± 0.13	-
C31	2-methyl-butyl-acetate	624-41-9	7.894	884	880	-	0.08 ± 0.10 ^b^	0.21 ± 0.05 ^a^
C32	heptanal	111-71-7	8.752	905	903	1.47 ± 0.64 ^a^	0.12 ± 0.14 ^b^	0.25 ± 0.08 ^b^
C33	methional	3268-49-3	9.000	912	907	0.85 ± 0.14 ^a^	0.15 ± 0.04 ^b^	0.15 ± 0.04 ^b^
C34	anisole	100-66-3	9.206	917	918	4.32 ± 2.47 ^a^	3.38 ± 1.44 ^a^	4.23 ± 1.68 ^a^
C35	benzaldheyde	100-52-7	10.755	959	961	1.08 ± 0.53 ^a^	0.35 ± 0.15 ^b^	1.03 ± 0.19 ^a^
C36	1-heptanol	111-70-6	11.100	969	967	-	0.08 ± 0.09 ^a^	0.27 ± 0.22 ^a^
C37	1-octen-3-ol	3391-86-4	11.584	982	980	6.77 ± 2.11 ^a^	0.66 ± 0.27 ^b^	0.48 ± 0.28 ^b^
C38	3-octanone	106-68-3	11.800	988	990	1.37 ± 0.48 ^a^	0.62 ± 0.19 ^b^	0.18 ± 0.01 ^c^
C39	3-octanol	589-98-0	12.124	997	994	-	0.41 ± 0.24 ^a^	0.20 ± 0.05 ^a^
C40	isobutyl-2-methylbutyrate	2445-67-2	12.300	1002	1009	-	0.30 ± 0.46	-
C41	octanal	124-13-0	12.300	1002	1003	0.34 ± 0.68 ^a^	-	0.62 ± 0.73 ^a^
C42	3-methyl-acid butanoic	589-59-3	12.500	1007	1004	-	-	-
C43	3-methylanisol	100-84-5	12.930	1020	1028	5.51 ± 1.32 ^a^	0.58 ± 0.49 ^b^	2.12 ± 2.46 ^ab^
C44	2-ethyl-1-hexanol	104-76-7	13.299	1031	1035	-	0.25 ± 0.16 ^a^	0.44 ± 0.34 ^a^
C45	benzeneacetaldehyde	122-78-1	13.709	1043	1047	3.49 ± 0.69 ^a^	0.46 ± 0.24 ^b^	1.51 ± 0.98 ^ab^
C46	1-octanol	111-87-5	14.288	1060	1067	-	0.07 ± 0.07 ^a^	0.18 ± 0.12 ^a^
C47	e-2-octenal	2548-87-0	14.581	1069	1062	2.08 ± 1.33 ^a^	0.02 ± 0.05 ^b^	0.09 ± 0.11 ^b^
C48	3-methyl-phenol	108-39-4	14.800	1075	1083	-	0.00 ± 0.01 ^a^	0.01 ± 0.02 ^a^
C49	2-nonanone	821-55-6	15.300	1090	1090	-	-	0.01 ± 0.01
C50	isoamyl-2methylbutyrate	27625-35-0	15.655	1100	1103	-	0.02 ± 0.04 ^a^	0.08 ± 0.06 ^a^
C51	nonanal	124-19-6	15.765	1104	1106	0.82 ± 0.17 ^a^	0.28 ± 0.27 ^a^	-
C52	2-methyl-butanoic acid	2445-78-5	15.765	1104	1105	-	0.41 ± 0.63 ^b^	1.27 ± 0.11 ^a^
C53	3-ethyl-5-methylphenol	698-71-5	16.001	1111	-	0.28 ± 0.12 ^a^	0.03 ± 0.08 ^a^	0.32 ± 0.47 ^a^
C54	benzeneethanol	60-12-8	16.015	1112	1113	0.06 ± 0.08 ^c^	0.25 ± 0.27 ^b^	0.66 ± 0.5 ^a^
C55	2,4,6-trimethyl-phenol	527-60-6	16.131	1115	-	-	-	-
C56	benzene, 1,2-dimethoxy-	91-16-7	17.118	1147	1146	0.71 ± 0.19 ^a^	0.97 ± 0.36 ^a^	1.30 ± 0.71 ^a^
C57	benzene, 1,3-dimethoxy-	151-10-0	17.694	1165	1182	1.00 ± 0.79 ^a^	0.01 ± 0.02 ^b^	0.09 ± 0.10 ^ab^
C58	benzene,1,4-dimethoxy	150-78-7	17.838	1169	1165	-	0.01 ± 0.02 ^a^	0.13 ± 0.17 ^a^
C59	caprylic acid	124-07-2	18.206	1181	1180	-	-	0.40 ± 0.32
C60	ethyl caprylate	106-32-1	18.700	1197	1196	-	-	0.02 ± 0.02
C61	2,4-nonadienal	5910-87-2	19.850	1236	1214	-	-	-
C62	3,2-dimethoxytoluene	4463-33-6	19.978	1241	-	0.10 ± 0.07 ^a^	0.01 ± 0.02 ^a^	0.03 ± 0.02 ^a^
C63	2,5-dimethoxytoluene	24599-58-4	20.295	1252	1259	0.31 ± 0.12 ^a^	0.05 ± 0.03 ^b^	0.06 ± 0.07 ^b^
C64	nonanoic acid	112-05-0	20.829	1270	1271	-	-	1.59 ± 1.89
C65	2-undecanone	112-12-9	21.412	1291	1296	0.34 ± 0.13 ^a^	0.05 ± 0.03 ^b^	0.03 ± 0.03 ^b^
C66	benzene,1,2,3-trimethoxy	634-36-6	22.010	1313	1315	0.01 ± 0.02 ^a^	0.01 ± 0.01 ^a^	0.00 ± 0.01 ^a^
C67	tetradecane	629-59-4	24.244	1398	-	-	-	0.03 ± 0.05
C68	2,4-bis(1,1-dimethylethyl)phenol	96-76-4	27.062	1512	1518	0.10 ± 0.06 ^a^	0.04 ± 0.02 ^a^	0.04 ± 0.04 ^a^
C69	tetradecanoic acid	554-63-8	33.258	1790	1763	-	-	-

RT = retention time. RI exp = retention index experimental. RI lit = retention index literature database NIS.

**Table 2:** Mean predicted scores (on a 1–9 scale) and standard deviation obtained in the sensory analysis (n = 16 for each cell). Different letters within the same column (sensory attribute) denote significant differences (*p* ≤ 0.05).

***Visual and texture descriptors***											
**Sample**	**Firmness**	**Shape**	**Uniformity**	**Peridium Colour**	**Peridium Shape**	**Gleba Colour**	**Gleba Sharpness**				
1_S1_T1	7.4 ± 10 ^a^	7.8 ± 1.0 ^a^	7.3 ± 1.6 ^a^	7.9 ± 0.8 ^a^	8.1 ± 0.6 ^a^	7.3 ± 1.3 ^ab^	6.5 ± 1.4 ^a^				
1_S1_T2	8.0 ± 0.70 ^a^	6.6 ± 1.5 ^ab^	4.8 ± 1.4 ^ab^	7.2 ± 1.5 ^a^	5.7 ± 1.5 ^b^	5.6 ± 1.3 ^ab^	5.9 ± 1.6 ^a^				
1_S1_T3	7.5 ± 1.2 ^a^	6.6 ± 1.5 ^ab^	5.6 ± 2.0 ^ab^	7.5 ± 0.7 ^a^	6.6 ± 1.8 ^ab^	6.9 ± 1.3 ^ab^	6.8 ± 1.4 ^a^				
1_S1_T4	6.1 ± 1.1 ^a^	6.8 ± 1.0 ^a^	5.8 ± 1.4 ^ab^	7.2 ± 1.2 ^a^	6.3 ± 1.4 ^ab^	7.8 ± 1.0 ^a^	6.8 ± 1.5 ^a^				
2_S1_T1	8.1 ± 0.7 ^a^	5.9 ± 1.0 ^ab^	5.3 ± 1.0 ^ab^	6.8 ± 1.0 ^a^	5.2 ± 1.5 ^b^	6.4 ± 1.5 ^ab^	4.8 ± 1.5 ^a^				
2_S1_T2	7.5 ± 1.2 ^a^	6.2 ± 1.0 ^ab^	5.5 ± 1.2 ^ab^	7.9 ± 0.6 ^a^	6.3 ± 0.9 ^b^	8.2 ± 0.7 ^a^	5.6 ± 2.1 ^a^				
2_S2_T1	6.9 ± 0.6 ^a^	6.4 ± 0.7 ^ab^	5.7 ± 1.4 ^ab^	7.1 ± 1.1 ^a^	6.8 ± 1.6 ^ab^	6.8 ± 1.2 ^ab^	5.1 ± 1.1 ^a^				
2_S2_T2	8.1 ± 0.8 ^a^	5.3 ± 1.2 ^ab^	3.7 ± 1.1 ^b^	7.1 ± 1.4 ^a^	5.8 ± 1.2 ^b^	5.0 ± 1.2 ^b^	6.9 ± 1.2 ^a^				
2_S2_T3	8.2 ± 0.7 ^a^	6.9 ± 1.1 ^ab^	4.9 ± 1.6 ^ab^	7.4 ± 1.4 ^a^	6.8 ± 0.9 ^ab^	8.3 ± 0.7 ^a^	8.2 ± 0.7 ^a^				
2_S2_T4	8.1 ± 0.9 ^a^	7.1 ± 0.7 ^a^	7.0 ± 1.0 ^a^	6.9 ± 1.5 ^a^	7.2 ± 1.1 ^ab^	7.5 ± 0.7 ^a^	6.4 ± 1.2 ^a^				
3_S1_T1	6.0. ± 1.4 ^a^	3.8 ± 1.8 ^b^	2.7 ± 1.3 ^b^	7.6 ± 1.2 ^a^	6.6 ± 1.6 ^ab^	7.8 ± 1.1 ^a^	5.5 ± 2.0 ^a^				
3_S1_T2	7.6 ± 0.7 ^a^	7.7 ± 0.9 ^a^	7.1 ± 1.3 ^a^	8.3 ± 0.6 ^a^	7.3 ± 0.7 ^ab^	8.3 ± 0.7 ^a^	6.5 ± 1.9 ^a^				
3_S2_T1	8.6 ± 0.5 ^a^	6.6 ± 1.0 ^ab^	5.9 ± 1.2 ^ab^	8.1 ± 0.7 ^a^	6.6 ± 1.5 ^ab^	7.9 ± 0.9 ^a^	7.8 ± 0.7 ^a^				
3_S2_T2	8.3 ± 0.6 ^a^	5.5 ± 1.4 ^ab^	5.4 ± 1.3 ^ab^	7.5 ± 1.2 ^a^	7.3 ± 1.6 ^ab^	8.1 ± 0.9 ^a^	6.9 ± 1.5 ^a^				
***Aromatic descriptors***											
**Sample**	**Equilibrium**	**Intensity**	**Complexity**	**Sulfur**	**Black olives**	**Mushroom**	**Leather**	**Butter**	**Fermentation**	**Nuts**	**Straw**
1_S1_T1	5.3 ± 1.3 ^a^	5.1 ± 1.5 ^a^	5.0 ± 1.4 ^a^	4.6 ± 1.6 ^ab^	4.4 ± 1.9 ^a^	3.1 ± 1.4 ^ab^	2.9 ± 1.6 ^a^	4.0 ± 2.0 ^a^	2.5 ± 2.2 ^a^	3.8 ± 2.1 ^a^	2.1 ± 1.5 ^a^
1_S1_T2	6.1 ± 1.3 ^a^	5.4 ± 1.5 ^a^	5.8 ± 1.0 ^a^	4.9 ± 1.7 ^ab^	3.9 ± 1.9 ^a^	2.9 ± 1.2 ^ab^	3.1 ± 2.1 ^a^	3.7 ± 1.9 ^a^	2.4 ± 2.0 ^a^	4.1 ± 1.8 ^a^	2.4 ± 2.0 ^a^
1_S1_T3	5.9 ± 1.1 ^a^	6.1 ± 1.4 ^a^	6.3 ± 0.9 ^a^	6.3 ± 1.3 ^a^	5.1 ± 2.0 ^a^	3.8 ± 1.9 ^ab^	3.4 ± 1.9 ^a^	4.1 ± 2.1 ^a^	2.6 ± 2.1 ^a^	4.3 ± 1.4 ^a^	2.4 ± 1.7 ^a^
1_S1_T4	5.3 ± 1.5 ^a^	4.9 ± 1.6 ^a^	4.8 ± 1.3 ^a^	5.0 ± 1.8 ^ab^	4.3 ± 2.4 ^a^	3.4 ± 1.7 ^ab^	3.1 ± 1.6 ^a^	3.1 ± 2.0 ^a^	2.5 ± 1.7 ^a^	3.6 ± 1.9 ^a^	2.3 ± 1.3 ^a^
2_S1_T1	5.9 ± 1.5 ^a^	5.4 ± 1.8 ^a^	5.7 ± 1.4 ^a^	4.2 ± 2.5 ^ab^	4.6 ± 1.8 ^a^	3.1 ± 1.7 ^ab^	2.7 ± 1.8 ^a^	3.6 ± 1.7 ^a^	2.1 ± 1.4 ^a^	3.1 ± 1.6 ^a^	2.1 ± 1.8 ^a^
2_S1_T2	6.6 ± 1.3 ^a^	7.1 ± 0.9 ^a^	6.6 ± 1.2 ^a^	5.9 ± 2.3 ^ab^	6.2 ± 1.9 ^a^	3.1 ± 1.4 ^ab^	2.9 ± 1.8 ^a^	5.8 ± 2.0 a	3.1 ± 2.1 ^a^	3.9 ± 1.4 ^a^	2.6 ± 2.1 ^a^
2_S2_T1	4.6 ± 1.7 ^a^	4.9 ± 1.1 ^a^	5.3 ± 1.5 ^a^	3.1 ± 1.5 ^b^	3.9 ± 1.8 ^a^	5.9 ± 2.0 ^a^	2.5 ± 2.0 ^a^	3.4 ± 1.7 ^a^	1.8 ± 1.2 ^a^	2.3 ± 1.7 ^a^	2.3 ± 1.9 ^a^
2_S2_T2	6.0 ± 1.5 ^a^	5.7 ± 1.1 ^a^	5.4 ± 1.2 ^a^	5.2 ± 1.9 ^ab^	4.9 ± 1.6 ^a^	4.0 ± 2.1 ^ab^	3.3 ± 1.3 ^a^	3.6 ± 1.6 ^a^	2.4 ± 1.5 ^a^	2.6 ± 1.9 ^a^	3.0 ± 2.0 ^a^
2_S2_T3	7.3 ± 0.9 ^a^	6.4 ± 1.3 ^a^	6.5 ± 1.2 ^a^	6.3 ± 2.2 ^ab^	5.9 ± 2.1 ^a^	3.5 ± 1.9 ^ab^	2.7 ± 2.1 ^a^	3.6 ± 1.6 ^a^	3.1 ± 1.5 ^a^	4.2 ± 2.0 ^a^	2.3 ± 1.3 ^a^
2_S2_T4	6.6 ± 1.5 ^a^	5.9 ± 1.5 ^a^	6.1 ± 1.7 ^a^	4.9 ± 2.3 ^ab^	5.1 ± 2.4 ^a^	2.6 ± 1.4 ^ab^	3.5 ± 1.7 ^a^	3.3 ± 2.1 ^a^	3.1 ± 1.7 ^a^	2.8 ± 1.6 ^a^	2.2 ± 0.8 ^a^
3_S1_T1	7.0 ± 1.5 ^a^	6.6 ± 2.0 ^a^	6.4 ± 1.9 ^a^	5.5 ± 2.1 ^ab^	5.1 ± 2.3 ^a^	2.9 ± 1.9 ^ab^	3.2 ± 1.7 ^a^	4.1 ± 2.4 ^a^	2.9 ± 1.7 ^a^	4.2 ± 2.2 ^a^	3.0 ± 2.1 ^a^
3_S1_T2	6.3 ± 1.7 ^a^	5.7 ± 1.7 ^a^	5.8 ± 1.8 ^a^	5.7 ± 2.0 ^ab^	5.2 ± 2.2 ^a^	2.6 ± 1.9 ^ab^	3.1 ± 2.2 ^a^	4.6 ± 1.7 ^a^	2.3 ± 1.5 ^a^	4.9 ± 1.9 ^a^	2.8 ± 1.9 ^a^
3_S2_T1	7.5 ± 0.6 ^a^	6.3 ± 1.5 ^a^	6.9 ± 1.2 ^a^	6.1 ± 2.3 ^ab^	5.8 ± 1.7 ^a^	2.0 ± 0.9 ^b^	2.6 ± 1.8 ^a^	4.3 ± 2.1 ^a^	3.1 ± 1.6 ^a^	4.6 ± 2.0 ^a^	2.4 ± 1.9 ^a^
3_S2_T2	4.4 ± 2.4 ^a^	6.0 ± 2.0 ^a^	4.9 ± 2.1 ^a^	4.3 ± 2.7 ^ab^	3.4 ± 2.4 ^a^	2.1 ± 1.7 ^b^	3.9 ± 1.8 ^a^	2.1 ± 1.7 ^a^	2.3 ± 2.0 ^a^	4.8 ± 2.2 ^a^	2.6 ± 2.0 ^a^

## Data Availability

The original contributions presented in the study are included in the article/Supplementary Materials, further inquiries can be directed to the corresponding author.

## Supplementary Materials

The following supporting information can be downloaded at: https://www.mdpi.com/article/10.3390/foods13060837/s1, Figure S1: Tucker-1 plot for attributes: (A) sulfur; (B) black olives; (C) mushroom; (D) leather. Samples correspond to those truffles used for training (more data shown in Figure 3). The points outside the circumference do not explain 100% of the data variance for the attribute model. Table S1: Attributes selected for visual phase in the sensory analysis. Values (low, medium, high) correspond to 1, 5, and 9 in a 9-point scale. Table S2: Correlation and *p* value values corresponding to Figure 2C. Table S3: VOCs odor and the possible relation with the sensory attributes.
