# Supplementary material for: Black Truffle Aroma Evaluation: SPME-GC-MS vs. Sensory Experts
Source: Foods. 2024 Mar 9;13(6):837. doi: 10.3390/foods13060837 (PMC10969277; doi:10.3390/foods13060837)
Supplement: Supplementary file 1 [file foods-13-00837-s001.zip › foods-2892462-supplementary.pdf]

Supplementary material

Black truffle aroma evaluation: SPME-GC-MS vs sensory experts

Eva Tejedor-Calvo[1,2,*] (etejedorc@aragon.es)**, Sergi García-Barreda**[1,2]**, Sergio Sánchez**[1,2]**, María Ángeles Sanz**[2,3]**, Pedro Marco**[1,2]

[1] Department of Plant Science, Agrifood Research and Technology Centre of Aragon (CITA), Av. Montañana, 930, 50059 Zaragoza, Spain

[2] Agrifood Institute of Aragón – IA2 (University of Zaragoza – CITA), 50018 Zaragoza, Spain

[3] Laboratories and Technological Assistance, Agrifood Research and Technology Centre of Aragon (CITA), Avda. Montãnana, 50059 Zaragoza, Spain

Table S1. Attributes selected for visual phase in the sensory analysis. Values (low, medium, high) correspond to 1, 5 and 9 in a 9-point scale.

Attributes	Low	Medium	High
Firmness	Gummy, Sagging	Slight springiness	Optimal truffle firmness
Shape	Irregular shape	Ovoid	Round
Uniformity	Heterogenous, with many, tall lobules	Few, small lobules	Homogeneous, without lobules
Peridium color	Red	-	Black
Peridium shape	Heterogenous		Homogeneous
Gleba color	White, grey	White/grey and black, brown	Black
Sharpness of the marbled pattern of the gleba	Blurred whitish veins or barely discernible	-	Well defined whitish veins

Table S2. Correlation and p value values corresponding to Figure 2C.

**Correlation values**											
	**Sulfur**	**Black**	**Mushroo**	**Leather**	**Butter**	**Fermente**	**Nuts**	**Straw**	**Equilibriu**	**Intensity**	**Complexit**
**Sulfur**	1	0.58363	-0.30722	-0.48058	0.3695	-0.234	-0.14513	-0.28825	0.27547	0.25925	0.082008
**Black**	0.58363	1	-0.15112	-0.25291	0.1953	-0.28912	-0.21345	-0.20865	0.33341	0.25466	0.26105
**Mushroom**	-	-0.15112	1	0.5446	-0.25375	0.21707	0.11094	0.23556	-0.15017	-	-0.12709
**Leather**	-	-0.25291	0.5446	1	-0.36064	0.23687	0.17616	0.31447	-0.086114	-	-0.029359
**Butter**	0.3695	0.1953	-0.25375	-0.36064	1	-0.13413	0.12739	-0.12345	0.16326	0.033846	0.021154
**Fermented**	-0.234	-0.28912	0.21707	0.23687	-0.13413	1	0.20945	0.32796	0.081879	0.074202	0.090968

**Nuts**	-	-0.21345	0.11094	0.17616	0.12739	0.20945	1	0.18134	0.080682	-	-
**Straw**	-	-0.20865	0.23556	0.31447	-0.12345	0.32796	0.18134	1	0.072489	-	0.11744
**Equilibriu**	0.27547	0.33341	-0.15017	-	0.16326	0.081879	0.080682	0.072489	1	0.16923	0.38545
**Intensity**	0.25925	0.25466	-0.006201	-	0.033846	0.074202	-0.047676	-0.039719	0.16923	1	0.47168
**Complexit**	0.08200	0.26105	-0.12709	-	0.021154	0.090968	-	0.11744	0.38545	0.47168	1

***p*-value**

	**Sulfur**	**Black**	**Mushroo**	**Leather**	**Butter**	**Fermente**	**Nuts**	**Straw**	**Equilibriu**	**Intensity**	**Complexit**
**Sulfur**	NA	0	1.04E-07	0	9.55E-11	6.08E-05	0.013688	6.48E-07	2.07E-06	8.31E-06	0.16513
**Black**	0	NA	0.010221	1.40E-05	0.0008620	5.97E-07	0.0002634	0.0003640	6.62E-09	1.21E-05	7.16E-06
**Mushroom**	1.04E-	0.010221	NA	0	1.31E-05	0.0002055	0.060052	5.41E-05	0.010713	0.91655	0.031066
**Leather**	0	1.40E-05	0	NA	2.84E-10	4.90E-05	0.0026989	4.97E-08	0.14491	0.62377	0.61977
**Butter**	9.55E-	0.0008620	1.31E-05	2.84E-10	NA	0.022809	0.030677	0.03626	0.0054838	0.56729	0.72073
**Fermented**	6.08E-	5.97E-07	0.0002055	4.90E-05	0.022809	NA	0.0003450	1.20E-08	0.1658	0.2093	0.1235
**Nuts**	0.01368	0.0002634	0.060052	0.002698	0.030677	0.0003450	NA	0.002003	0.1721	0.42023	0.91334
**Straw**	6.48E-	0.0003640	5.41E-05	4.97E-08	0.03626	1.20E-08	0.002003	NA	0.22003	0.50197	0.046461
**Equilibriu**	2.07E-	6.62E-09	0.010713	0.14491	0.0054838	0.1658	0.1721	0.22003	NA	0.003973	1.23E-11
**Intensity**	8.31E-	1.21E-05	0.91655	0.62377	0.56729	0.2093	0.42023	0.50197	0.0039735	NA	0
**Complexit**	0.16513	7.16E-06	0.031066	0.61977	0.72073	0.1235	0.91334	0.046461	1.23E-11	0	NA

Table S3. VOCs odor and the possible relation with the sensory attributes.

**Name**	**CAS**	**Odor***	**Attribute selected by the trained panel**
Ethanol	64-17-5	sweet	ni
Dimethyl sulfide	75-18-3	cabbage, sulfur, gasoline	sulfur; black olives
1-propanol	71-23-8	alcohol, pungent	fermentation
Propanal-2-methyl	78-84-2	pungent, malt, green	fermentation
Butanal	123-72-8	pungent, green	ni
3-methyl-2-butanone	563-80-4	camphor	leather
2-butanone	78-93-3	ether	ni
2-methyl-1-propanol	78-83-1	wine, solvent, bitter	fermentation
butanal-3-methyl	590-86-3	malt	fermentation
butanal-2-methyl	96-17-3	cocoa, almond	nuts
2-pentanone	107-87-9	ether, fruit	ni
Pentanal	110-62-3	almond, malt, pungent	nuts; fermentation
3-methyl-1-butanol	123-51-3	whiskey, malt, burnt	fermentation
2-methyl-1-butanol	137-32-6	wine, onion	fermentation
Dimethyl-disulfide	624-92-0	onion, cabbage, black olives	sulfur; black olives
Isobutylacetate	110-19-0	fruit, apple, banana	ni
Hexanal	66-25-1	grass, tallow, fat	straw
ethyl-2-methylbutanoate	7452-79-1	apple	ni
ethyl-3-methylbutanoate	108-64-5	fruit	ni
Hexanol	111-27-3	resin, flower, green	ni
Isoamylacetate	123-92-2	banana	ni
2-methyl-butyl-acetate	624-41-9	fruit	ni
Heptanal	111-71-7	fat, citrus, rancid	butter
Methional	3268-49-3	cooked potato	ni
Benzaldheyde	100-52-7	almond, burnt sugar	nuts
1-heptanol	111-70-6	chemical, green	straw
1-octen-3-ol	3391-86-4	mushroom	mushroom

3-octanone	106-68-3	herb, butter, resin	butter
3-octanol	589-98-0	moss, nut, mushroom	mushroom; nuts
Octanal	124-13-0	fat, soap, lemon, green	butter
2-ethyl-1-hexanol	104-76-7	rose, green	ni
Benzeneacetaldehyde	122-78-1	hawthorne, honey, sweet	ni
1-octanol	111-87-5	chemical, metal, burnt	ni
E-2-octenal	2548-87-0	green, nut, fat	nuts
3-methyl-phenol	108-39-4	fecal, plastic	leather
2-nonanone	821-55-6	hot milk, soap, green	ni
Nonanal	124-19-6	fat, citrus, green	ni
Benzeneethanol	60-12-8	honey, spice, rose, lilac	ni
caprylic acid	124-07-2	sweat, cheese	butter
ethyl caprylate	106-32-1	fruit, fat	butter
2,4-nonadienal	5910-87-2	fat, wax, green	butter
nonanoic acid	112-05-0	green, fat	butter
2-undecanone	112-12-9	orange, fresh, green	ni

ni – not identified

* Odor data was reported by flavornet website and some authors (Campo et al., 2017; Culleré et al., 2017; Tejedor-Calvo et al., 2021; Tejedor-Calvo, García-Barreda, et al., 2023).

Figure S1. Tucker-1 plot for attributes: A) sulfur; B) black olives; C) mushroom; D) leather. Samples correspond to those truffles for training (more data shown in Figure 3). The points outer the circumference does not explain 100% of the data variance for the attribute model.

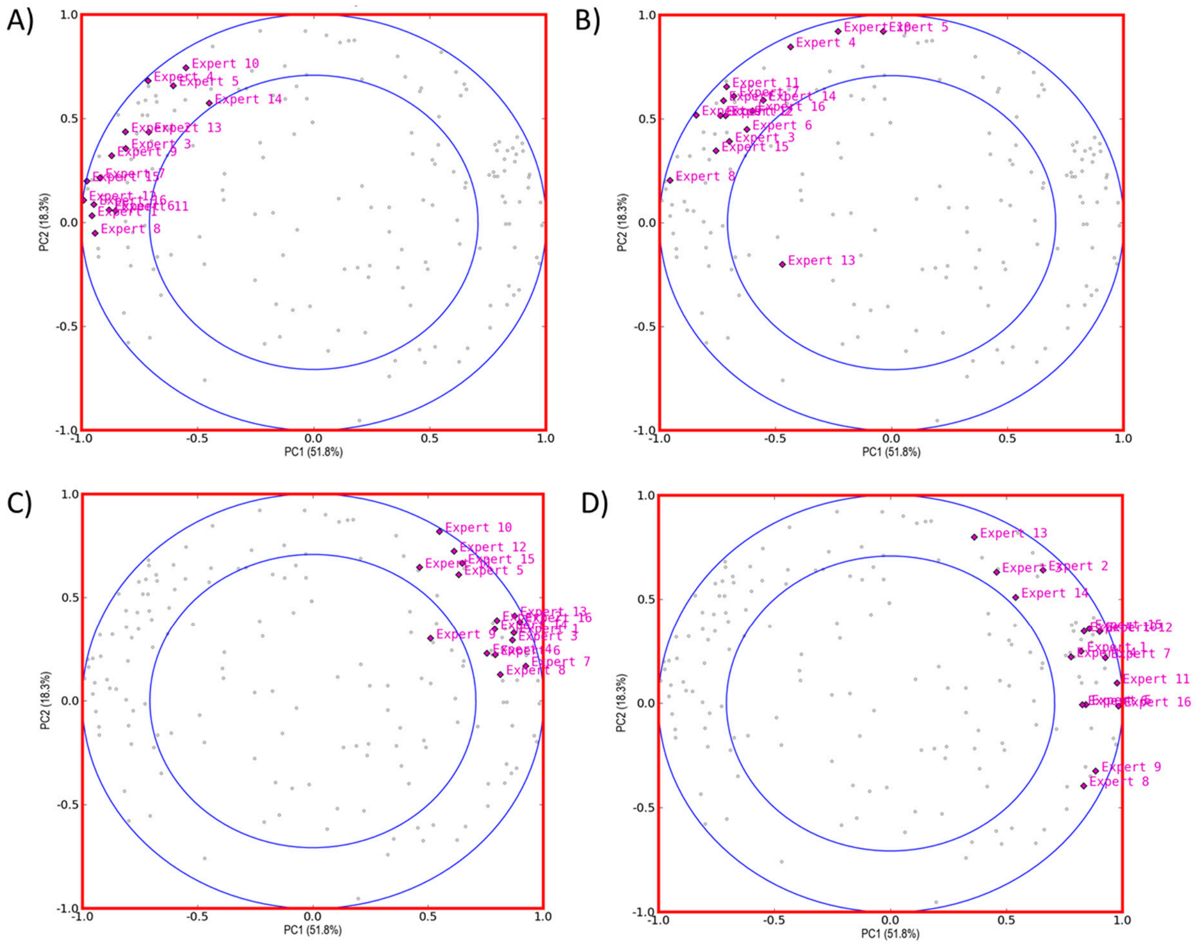
A)
B)
C)
D)
PC1 (51.8%)
PC2 (18.3%)
-1.0
-0.5
0.0
0.5
1.0
